# Nasal irrigation with corticosteroids in Brazil: the clinical response of 1% compounded budesonide drops and betamethasone cream

**DOI:** 10.1016/j.bjorl.2021.06.008

**Published:** 2021-08-04

**Authors:** Gabriela Ricci Luz-Matsumoto, Erika Cabernite-Marchetti, Layla Sayuri Kaczorowski Sasaki, Germana Jardim Marquez, Laura Schmitt de Lacerda, Thiago Ribeiro de Almeida, Eduardo Macoto Kosugi

**Affiliations:** Universidade Federal de São Paulo (UNIFESP), Escola Paulista de Medicina (EPM), Setor de Rinologia, São Paulo, SP, Brazil

**Keywords:** Sinusitis, Corticosteroids, Nasal irrigation, Nasal spray

## Abstract

**Introduction:**

High-volume corticosteroid nasal irrigation is a treatment option in patients with chronic rhinosinusitis. In Brazil, alternatives are used to optimize its cost and popularize its use, such as 1% compounded budesonide drops or betamethasone cream, and it is necessary to study these treatment modalities.

**Objective:**

To evaluate the clinical response of nasal irrigation with 1% compounded budesonide drops or betamethasone cream compared to nasal sprays utilized in patients with chronic rhinosinusitis.

**Methods:**

This was a retrospective observational study with 257 patients. One hundred and eight patients using corticosteroid nasal irrigation (292 treatment cycles) and 149 using corticosteroid nasal spray (300 treatment cycles) were included. Evaluation of subjective improvement, adverse events, exacerbations, and objective assessments with SNOT-22 and Lund–Kennedy endoscopic score were performed, in addition to sub-analyses related to nasal polyps and previous surgery.

**Results:**

Corticosteroid nasal irrigation and corticosteroid nasal spray improved the Lund–Kennedy endoscopic score, with more adverse events in the corticosteroid nasal irrigation group. Previous surgery increased corticosteroid nasal irrigation improvement, with greater subjective improvement and fewer exacerbations. 1% compounded budesonide drops were better than betamethasone cream in the Lund–Kennedy endoscopic score, with fewer adverse events. A 1,000 µg dose of 1% compounded budesonide drops was more effective than 500 µg.

**Conclusion:**

Corticosteroid nasal irrigation was effective in improving the Lund–Kennedy endoscopic score in chronic rhinosinusitis, especially in patients with nasal polyps and previous surgery, in addition to promoting a higher rate of subjective improvement and fewer exacerbations than corticosteroid nasal spray, but with more adverse events. 1% compounded budesonide drops improved the Lund–Kennedy endoscopic score with fewer adverse events than betamethasone cream, particularly at higher doses (1000 µg).

## Highlights

• Nasal irrigation with corticosteroids is effective in chronic rhinosinusitis.
• Irrigation with corticosteroids is more effective in patients with nasal polyps and previous sinus surgery.
• 1% compounded budesonide drops are better than betamethasone cream.
• Higher doses of compounded 1% budesonide drops are more effective than lower ones.

## Introduction

There is an increasing perception that chronic rhinosinusitis (CRS) is not a single disease that can be classified solely by the presence or absence of nasal polyps (CRSwNP and CRSnNP, respectively),^1^ but rather as an umbrella term that encompasses several different diseases, with distinct pathophysiological mechanisms, leading to chronic inflammation and similar symptoms.^2^ Therefore, their therapeutic results can also be very different, ranging widely from poor response to the cure. The focus of CRS treatment is to control inflammation and reduce infectious exacerbations.^1^ To achieve clinical disease control, multiple therapeutic strategies may be required, especially topical therapies and surgical interventions.^3^ Systemic corticosteroids (CS) should not be routinely used in CRS management, due to unwanted side effects.^3^, ^4^

Topical corticosteroid nasal spray (CSNS) therapy and nasal lavage with saline solution have their benefits well established in the literature.^3^, ^4^ Despite the high level of recommendation for CSNS use in the treatment of CRS,^1^, ^3^, ^4^ it is known that this treatment can have failures, which is why high-volume corticosteroid nasal irrigation (CSNI) started to be recommended, aiming to increase sinus penetration and, consequently, increase the drug concentration in the diseased mucosa.^5^ CSNI has shown to be effective in post-surgical patients, in whom the drainage ostia have been enlarged and in those with difficult-to-treat CRS.^2^, ^5^, ^6^, ^7^ It is known that, even in individuals not submitted to surgery (and without polyps), nasal irrigation has greater sinus penetration than nasal sprays,^8^ although less than operated sinuses.^9^

In Brazil, alternatives are used to optimize the cost of CSNI, such as 0.5 mg/g betamethasone cream or 1% compounded budesonide drops,^2^ to be mixed with saline solution and applied to the nasal cavity.^2^ These alternatives allowed the popularization of CSNI in our population. There are no robust data on the effectiveness of these alternative CSNI modalities, highlighting the need to evaluate these methods.

The aim of this study was to evaluate the clinical response of nasal irrigation with 1% compounded budesonide drops or betamethasone cream in comparison to nasal sprays and the influence factors in patients with CRS treated at an outpatient clinic specialized in Rhinology.

## Methods

### Ethical considerations

This study was approved by the Research Ethics Committee of our institution under number 1166/2017. Written free and informed consent was obtained from all patients who agreed to participate.

### Study population

All electronic medical records of patients treated in Rhinology Section of Sao Paulo Hospital (HU-HSP), Federal University of Sao Paulo (UNIFESP) - Escola Paulista de Medicina (EPM) between January 2013 and December 2019 were evaluated, and 378 treated patients with a confirmed diagnosis of CRS were selected. Of these, 257 patients had nasal topical corticosteroid treatment cycles with complete pre- and post-evaluation data and were included in this study, 205 patients with CRSwNP and 52 with CRSnNP.

### Inclusion criteria

• Aged >18 years;
• Confirmed diagnosis of CRS according to the American Academy of Rhinology criteria^3^;
• Treatment cycles between 3 and 6 months of CSNI or CSNS;
• Complete subjective and objective evaluation data, pre- and post-treatment cycle, between January 2013 and December 2019.

### Exclusion criteria

Use of oral, intramuscular or intravenous corticosteroids during the studied cycle.

### Study design

A retrospective observational study (historical cohort) was carried out with two groups: CSNI and CSNS. The CSNI group had 108 patients who were submitted to at least one treatment cycle with CSNI (totaling 292 treatment cycles with complete data), which were compared to 149 patients who only underwent treatment cycles with CSNS (totaling 300 treatment cycles with complete data) in the CSNS group.

The treatment cycle was defined as a period of 3–6 months with topical nasal treatment with CS, with subjective and objective assessment at the beginning and at the end of this period recorded in electronic medical records. Patients in the CSNS group were treated with nasal CS in commercial spray formulations; while those in the CSNI group were treated with 1% compounded budesonide drops (each drop presented 500 µg of budesonide) or betamethasone cream (0.5 mg/g betamethasone) that were diluted in 250 mL of alkaline homemade saline solution (250 mL of water + 1 level teaspoon of table salt + 1 level teaspoon of sodium bicarbonate).

Epidemiological data such as gender and age were collected; associated diseases such as asthma, acetylsalicylic acid (ASA) intolerance, non-steroidal anti-inflammatory (NSAID) intolerance, dipyrone intolerance, NSAID-exacerbated respiratory disease (N-ERD), allergic fungal rhinosinusitis (AFRS); history such as current smoking, former smoking and previous endoscopic sinus surgery (ESS) for CRS; main complaint; and details on the topical therapy used (drug, dose, volume, time of use, adverse events).

The subjective assessment of symptom improvement during the cycle was followed by the investigation of adverse events, CRS exacerbations, need for surgery after the treatment, and systemic medications used.

The patients’ objective clinical evaluation was performed with the 22-item SinoNasal Outcome Test test (SNOT)-22^10^ and the Lund–Kennedy Endoscopic Score (LKES)^11^ applied pre- and post-treatment.

Sub-analyses were performed separating patients with CRSwNP and CRSnNP, previously submitted to ESS and those not submitted to ESS, in addition to sub-analyses comparing the alternative CSNI therapies (1% compounded budesonide drops and betamethasone cream).

### Statistical analysis

Continuous variables were represented by mean and standard deviation. Categorical variables were represented by absolute frequencies (n) and percentages (%). Data distribution was analyzed using the Kolmogorov–Smirnov test. The Z-score was used to normalize the non-parametric distribution data. The comparison of continuous data was performed using Student’s *t* test. Pearson’s Chi-Square Test or Fisher’s Exact Test was used to compare the frequency between groups. The significance level was set at *p* ≤ 0.05. The SPSS version 24.0 software was used in the analyses.

## Results

### Clinical and epidemiological characteristics of CRSwNP vs. CRSnNP

The mean age of the total sample was 55.5 years (±13.2), with a predominance of women in patients with CRSnNP (69.2% vs. 49.3%; *p* = 0.01*). There was an uneven distribution between the groups regarding the main complaint, with a predominance of anterior rhinorrhea (30.1% vs. 14.2%; *p* = 0.005*) and facial pain (21.2% vs. 5.4%; *p* = 0.001*) in patients with CRSnNP, and a predominance of hyposmia (33.7% vs. 7.7%; *p* = 0.002*) in patients with CRSwNP. As for the comorbidities, there was an uneven distribution of the presence of asthma (42.4% vs. 9.6%; *p* < 0.0001*), ASA intolerance (14.6% vs. 1.9%; *p* = 0.01*), dipyrone intolerance (13.7% vs. 1.9%; *p* = 0.02*) and N-ERD (13.7% vs. 1.9%; *p* = 0.02*), with a predominance in patients with CRSwNP. The distribution data are shown in Table 1.

**Table 1:** Characteristics of patients according to the presence of polyps.

Characteristic		Type of chronic rhinosinusitis						Test	*p*-Value
		CRSwNP		CRSnNP		Total			
		n = 205		n = 52		n = 257			
Age (years)	M/SD	55.2	13.4	56.9	12.4	55.5	13.2	T	0.38
Female gender	n (%)	101	49.3	36	69.2	137	53.3	Chi	0.01^a^
Main symptom									
Nasal obstruction	n (%)	78	38.1	19	36.5	97	37.7	Chi	1
Anterior rhinorrhea	n (%)	29	14.2	16	30.1	45	17.5	Chi	0.005^a^
Posterior rhinorrhea	n (%)	14	6.8	2	3.9	16	6.2	Chi	0.54
Hyposmia	n (%)	69	33.7	4	7.7	73	28.4	Chi	0.0002^a^
Facialgia	n (%)	11	5.4	11	21.2	22	8.6	Fisher	0.001^a^
Irritation symptoms	n (%)	4	2.0	0	0.0	2	0.8	Fisher	0.59
Comorbidities									
Asthma	n (%)	87	42.4	5	9.6	92	35.8	Chi	0.0001^a^
ASA intolerance	n (%)	30	14.6	1	1.9	31	12.1	Chi	0.01^a^
NSAID intolerance	n (%)	19	9.3	1	1.9	20	7.8	Fisher	0.09
Dipyrone intolerance	n (%)	28	13.7	1	1.9	29	11.3	Chi	0.02^a^
NERD	n (%)	28	13.7	1	1.9	29	11.3	Chi	0.02^a^
AFRS	n (%)	2	1.0	0	0.0	2	0.8	Fisher	1
Active smoker	n (%)	12	5.9	4	7.7	16	6.2	Fisher	0.75
Ex-smoker	n (%)	25	12.2	7	13.5	32	12.5	Chi	0.81
Previous ESS	n (%)	131	63.9	31	59.6	162	63.0	Chi	0.57
CSNI	n (%)	87	42.4	21	40.4	108	42.0	Chi	0.79
CSNS	n (%)	118	57.6	31	59.6	149	58.0	Chi	0.79

CRSwNP, chronic rhinosinusitis with nasal polyps; CRSnNP, chronic rhinosinusitis without nasal polyps; M, mean; SD, standard deviation; n, number; %, percentage; ASA, acetylsalicylic acid; NSAIDs, non-steroidal anti-inflammatory drugs; NERD, NSAID-exacerbated respiratory disease; AFRS, allergic fungal rhinosinusitis; ESS, endoscopic sinus surgery; CSNI, corticosteroid nasal irrigation; CS, corticosteroid.

a p < 0.05.

The initial patient assessment showed that the CRSnNP group had higher mean SNOT-22 scores than the CRSwNP group (39.5 vs. 29.6 points; *p* = 0.0003*). The questions with the greatest impact on the quality of life in the CRSnNP group were number 5 (Postnasal discharge; 2.53), number 1 (Need to “blow” nose; 2.32) and number 22 (Blockage/congestion of nose; 2.29), while in the CRSwNP group we identified number 21 (Sense of taste/smell; 2.54), number 5 (Postnasal discharge; 2.14) and number 1 (Need to “blow” the nose; 1.77). All questions showed an uneven distribution between the groups, except for questions 2, 3, 7 and 18.

The patients’ initial mean LKES was 4.7 with a standard deviation (SD) of 3.1. The CRSwNP group had higher scores, partly due to the presence of polyps that increase the LKES (5.0 vs. 3.0; *p* < 0.0001*). However, even removing the values ​​of polyps in the LKES, CRSwNP patients have significantly higher scores than CRSnNP (3.5 vs. 3.0; *p* = 0.04*). Patients with CRSwNP had a higher proportion of grade 2 (polypoid) edema (31.4% vs. 13.1%; *p* < 0.0001*); while those with CRSnNP showed a higher proportion of grade 2 (purulent) secretion (23.8% vs. 11.5%; *p* < 0.0001*). The pre-treatment impact of the disease (CRSwNP and CRSnNP) measured by the SNOT-22 and by the LKES can be analyzed in Table 2.

**Table 2:** Pre-treatment impact of the disease between the CRSwNP and CRSnNP groups.

Disease characteristics		Type of chronic rhinosinusitis						Test	*p*-Value
		CRSwNP		CRSnNP		Total			
		n = 205		n = 52		n = 257			
**Pre-treatment SNOT-22 (BR)**		**Mean**	**SD**	**Mean**	**SD**	**Mean**	**SD**		
1	Need to “blow” one’s nose	1.8	1.5	2.3	1.5	1.9	1.6	T	0.002^a^
2	Sneezing	1.5	1.4	1.8	1.5	1.6	1.4	T	0.19
3	“Runny” nose	1.4	1.5	1.5	1.5	1.4	1.5	T	0.33
4	Coughing	1.2	1.5	2.0	1.8	1.4	1.6	T	<0.001^a^
5	Secretion from the nose going to the throat	2.1	1.7	2.5	1.8	2.2	1.7	T	0.05^a^
6	Thick secretion coming out of the nose	1.6	1.7	2.0	1.7	1.7	1.7	T	0.03^a^
7	Sensation of aural fullness or clogged ears	1.4	1.7	1.7	1.9	1.5	1.7	T	0.16
8	Dizziness or vertigo	0.9	1.3	1.5	1.7	1.0	1.4	T	0.001^a^
9	Earache	0.5	1.0	0.7	1.2	0.5	1.1	T	0.04^a^
10	Facial pain or pressure	1.0	1.5	1.8	1.8	1.2	1.6	T	<0.001^a^
11	Difficulty falling asleep	1.3	1.7	1.9	1.8	1.4	1.8	T	0.002^a^
12	Waking up in the middle of the night	1.3	1.7	1.8	1.8	1.4	1.7	T	0.03^a^
13	Lack of a good night’s sleep	1.4	1.8	2.1	2.0	1.5	1.8	T	0.001^a^
14	Waking up tired	1.2	1.7	1.8	1.8	1.3	1.7	T	0.004^a^
15	Fatigue or tiredness during the day	1.2	1.7	1.8	1.9	1.3	1.7	T	0.003^a^
16	Decrease in one’s performance to carry out the activities of daily living	1.1	1.6	1.9	1.8	1.2	1.6	T	<0.001^a^
17	Decrease in one’s concentration to carry out the activities of daily living	1.1	1.5	1.7	1.7	1.2	1.6	T	<0.001^a^
18	Frustrated, agitated or angry	1.4	1.8	1.8	2.0	1.5	1.8	T	0.07
19	Sadness	1.0	1.6	1.6	1.9	1.1	1.7	T	0.004^a^
20	Feeling of shame	1.0	1.6	1.5	1.9	1.1	1.7	T	0.01^a^
21	Difficulty in feeling “smells” or “tastes”	2.5	1.9	1.9	1.9	2.4	1.9	T	0.005^a^
22	Stuffy nose	1.8	1.7	2.3	1.8	1.8	1.8	T	0.008^a^
Total		29,6	23.1	39.5	24.7	31.3	23.6	T	<0.001^a^
**Pre-treatment LKES per nasal fossa**									
**Per nasal fossa**		**n**	**%**	**n**	**%**	**n**	**%**		
Edema	Grade 0	263	28.9	95	46.1	358	30.2	Chi	<0.001^a^
	Grade 1	408	41.7	84	40.8	492	41.6		
	Grade 2	307	31.4	27	13.1	334	28.2		
Secretion	Grade 0	402	41.1	86	41.8	488	41.2	Chi	<0.001^a^
	Grade 1	464	47.4	71	34.5	535	45.2		
	Grade 2	112	11.5	49	23.8	161	13.6		
**Total pre-treatment LKES**		**Mean**	**SD**	**Mean**	**SD**	**Mean**	**SD**		
Polyps + edema + secretion		5,0	3.2	3.0	2.3	4.7	3.1	T	<0.001^a^
Edema + secretion (without polyp score)		3,5	2.3	3.0	2.3	3.4	2.3	T	0.04^a^

CRSwNP, chronic rhinosinusitis with nasal polyps; CRSnNP, chronic rhinosinusitis without nasal polyps; LKES, Lund-Kennedy endoscopic score; SNOT-22 (BR), SinoNasal Outcome Test-22 in Brazilian Portuguese; SD, standard deviation; n, number; %, percentage.

a p < 0.05.

### Comparison of CSNI vs. CSNS on the total sample

Considering now the treatment cycles, 592 cycles were counted, being 292 of CSNI and 300 of CSNS. In the 292 cycles of CSNI, the most often used corticosteroid was 1% compounded budesonide drops (51% of the cycles), followed by betamethasone cream (49%). The most often used daily dose of 1% compounded budesonide drops was 1000 µg (48.3%), followed by 500 µg (44.3%), diluted in 250 mL of saline solution. For the cream, 0.5 mg of betamethasone dipropionate (0.5 mg/g) was used, diluted in this same daily volume of saline solution. All patients used a syringe as an irrigation device. In the 300 cycles of CSNS, the most often used drug was budesonide (90% of the cycles), most of which (68.7%) at a dose of 400 µg per day. In addition, 89.7% of the CSNS cycles were accompanied by nasal irrigation with saline solution concomitantly.

There was a statistically significant difference in the epidemiological characteristics between patients who underwent cycles of CSNI and CSNS, with less female predominance (44.2% vs. 55.0%; *p* = 0.008*) and younger mean age (55.2 years vs. 57.7 years; *p* = 0.02*) in the CSNI group. Additionally, patients undergoing CSNI cycles had more comorbidities: dipyrone intolerance (15,4% vs. 9,0%; *p* = 0,02*) and N-ERD (14.7% vs. 9.7%; *p* = 0.06); and more co morbid antecedents: current smoking (12.0% vs. 3.7%; *p* = 0.0001*) and previous ESS (83.6% vs. 52.7%; *p* < 0.0001). There were no other initial differences, including the presence of nasal polyps (83.2% vs. 82.0%; *p* = 0.70).

The results of theCSNI and CSNS cycles in all patients are shown in Table 3. There was a significant improvement in LKES with both treatments (Irrigation: 4.8–4.4; *p* = 0.01*; spray: 4.6–4.3; *p* = 0.02*), but with more adverse events during CSNI cycles (2.4% vs. 0.3%; *p* = 0.04*). Ear fullness (2 patients), epistaxis (2), nasal irritation (1), epigastric pain (1) and nausea (1) were the adverse events that occurred with CSNI, while CSNS caused epistaxis in only one patient. CSNI showed to be more effective to prevent exacerbations than CSNI (6.5% vs. 10.7%; *p* = 0.07) but did not change the surgical indication rate after treatment (11.3% vs. 12.0%; *p* = 0.79).

**Table 3:** Results of CSNI and CSNS treatments for all patients.

All patients		Treatment cycle						Test	*p*-Value
		CSNI		CSNS		Total			
		n = 292		n = 300		n = 592			
Time of treatment (days)	M/SD	150.7	98.5	149.1	71.7	149.9	85.9	T	0.82
Subjective improvement	n (%)	241	82.5	230	76.7	471	79.6	Chi	0.08
Pre-treatment SNOT-22	M/SD	32.8	23.8	29.8	23.4	31.3	23.6	T	0.13
Post-treatment SNOT-22	M/SD	31.2	24.9	28.9	24.4	30.0	24.6	T	0.27
		Pre-post T	0.06	Pre-post T	0.19	Pre-post T	0.04^a^		

Pre-treatment total LKES	M/SD	4.8	3.0	4.6	3.2	4.7	3.1	T	0.41
Post-treatment total LKES	M/SD	4.4	3.2	4.3	3.1	4.3	3.2	T	0.53
		Pre-post T	0.01^a^	Pre-post T	0.02^a^	Pre-post T	0.001^a^		

Adverse events	n (%)	7	2.4	1	0.3	8	1.4	Fisher	0.04^a^
Exacerbations	n (%)	19	6.5	32	10.7	51	8.6	Chi	0.07
Need for subsequent surgery	n (%)	33	11.3	36	12.0	69	11.7	Chi	0.79

Chi, Chi-square test; CSNI, corticosteroid nasal irrigation; CSNS, Topical corticosteroid nasal spray; Fisher, Fisher exact test; M, mean; SD, standard deviation; n, number; %, percentage; LKES, Lund–Kennedy endoscopic score; SNOT-22 (BR), SinoNasal Outcome Test-22 in Brazilian Portuguese; T, T-test.

### Comparison of CSNI vs. CSNS according to the presence or absence of nasal polyps

When considering only CRSwNP, we obtained 489 treatment cycles, 243 of CSNI and 246 of CSNS. Patients with CRSwNP undergoing CSNI cycles showed a lower female predominance (41.2% vs. 51.2%; *p* = 0.03*), younger mean age (55.1 vs. 57.5; *p* = 0.04*), higher proportion of dipyrone intolerance (18.2% vs. 11.0%; *p* = 0.03*), of N-ERD (17.7% vs. 11.4%; *p* = 0.05*), higher proportion of current smoking (11.5% vs. 3.7%; *p* = 0.001*) and higher proportion of previous ESS (85.2% vs. 52.4%; *p* < 0.0001*) than those submitted to CSNS cycles.

The results of the CSNI and CSNS cycles only in patients with CRSwNP were shown in Table 4. There was a significant improvement in the LKES for both cycles (Irrigation: 5.1–4.8; *p* = 0.04* and spray: 4.9–4.6; *p* = 0.03*) with a tendency towards more adverse events (2.5% vs. 0.4%; *p* = 0.07) and fewer exacerbations (6.2% vs. 10 .6%; *p* = 0.08) in the CSNI group than in the CSNS.

**Table 4:** Results of CSNI and CSNS treatments for patients with CRSwNP.

Patients with CRSwNP		Treatment cycle						Test	*p*-value
		CSNI		CSNS		Total			
		n = 243		n = 246		n = 489			
Time of treatment (days)	M/SD	154.5	102.2	149.6	72.7	152.0	88.5	T	0.54
Subjective improvement	n (%)	205	84.4	193	78.5	398	81.4	Chi	0.09
Pre-treatment SNOT-22	M/SD	31.6	23.6	27.7	22.4	29.6	23.1	T	0.06
Post-treatment SNOT-22	M/SD	30.0	24.9	26.6	23.2	28.3	24.1	T	0.12
		Pre-post T	0.09	Pre-post T	0.17	Pre-post T	0.05^a^		
Pre-treatment total LKES	M/SD	5.1	3.0	4.9	3.3	5.0	3.2	T	0.42
Post-treatment total LKES	M/SD	4.8	3.2	4.6	3.2	4.7	3.2	T	0.44
		Pre-post T	0.04^a^	Pre-post T	0.03^a^	Pre-post T	0.005^a^		
Adverse events	n (%)	6	2.5	1	0.4	8	1.6	Fisher	0.07
Exacerbations	n (%)	15	6.2	26	10.6	41	8.4	Chi	0.08
Need for subsequent surgery	n (%)	28	11.5	30	12.2	58	11.9	Chi	0.82

Chi, Chi-square test; CSNI, corticosteroid nasal irrigation; CSNS, Topical corticosteroid nasal spray; Fisher, Fisher exact test; M, mean; SD, standard deviation; n, number; %, percentage; LKES, Lund–Kennedy endoscopic score; SNOT-22 (BR), SinoNasal Outcome Test-22 in Brazilian Portuguese; T, T-test.

a p < 0.05.

There were only 103 cycles of treatments in patients with CRSnNP (49 cycles of CSNI and 54 of CSNS), with no difference regarding patient characteristics between the cycles, except for more patients with previous ESS in the CSNI group (75.5% vs. 53.7%; *p* = 0.02*). There were also no differences regarding the results between the cycles.

### Comparison of CSNI vs. CSNS according to previous surgical status

When considering only patients with previous ESS, 402 treatment cycles were analyzed, being 244 of CSNI and 158 of CSNS. Patients with previous ESS undergoing CSNI cycles showed a lower female predominance (40.6% vs. 51.3%; *p* = 0.04*), younger mean age (54.3 vs. 57.6; *p* = 0.008*) and more current smoking (13.9% vs. 5.7%; *p* = 0.009*), with no differences in the other parameters, including the presence of polyps (84.8% vs. 81.7%; *p* = 0.40*). As for the results of the cycles, patients with previous ESS undergoing CSNI cycles had a higher rate of subjective improvement (85.3% vs. 77.2%; *p* = 0.04*) and a lower rate of exacerbations (6.6 % vs. 13.3%; *p* = 0.02*) than those submitted to CSNS cycles, and only those submitted to CSNI cycles showed improvement in LKES (4.7–4.3; *p* = 0, 01*), as shown in Table 5.

**Table 5:** Results of CSNI and CSNS treatments for patients previously submitted to ESS.

Patients with previous ESS		Treatment cycle						Test	*p*-Value
		CSNI		CSNS		Total			
		n = 244		n = 158		n = 402			
Treatment time (days)	M/SD	150.8	96.3	150.8	70.5	150.8	87.0	T	0.99
Subjective improvement	n (%)	208	85.3	122	77.2	330	82.1	Chi	0.04^a^
Pre-treatment SNOT-22	M/SD	30.5	23.5	28.6	23.6	29.8	23.5	T	0.42
Post-treatment SNOT-22	M/SD	29.5	25.0	28.5	24.1	29.1	24.7	T	0.69
		Pre-post T	0.18	Pre-post T	0.47	Pre-post T	0.22		
Pre-treatment total LKES	M/SD	4.7	3.0	3.8	3.3	4.3	3.1	T	0.01^a^
Post-treatment total LKES	M/SD	4.3	3.2	3.8	3.1	4.1	3.2	T	0.19
		Pre-post T	0.01^a^	Pre-post T	0.40	Pre-post T	0.05^a^		
Adverse events	n (%)	7	2.9	1	0.6	8	2.0	Fisher	0.16
Exacerbations	n (%)	16	6.6	21	13.3	37	9.2	Chi	0.02^a^
Need for subsequent surgery	n (%)	22	9.0	12	7.6	34	8.5	Chi	0.62

Chi, Chi-square test; CSNI, corticosteroid nasal irrigation; CSNS, Topical corticosteroid nasal spray; Fisher, Fisher exact test; M, mean; SD, standard deviation; n, number; %, percentage; LKES, Lund–Kennedy endoscopic score; SNOT-22 (BR), SinoNasal Outcome Test-22 in Brazilian Portuguese; T, T-test.

a p < 0.05.

In patients never submitted to ESS, 190 treatment cycles were performed (48 of CSNI and 142 of CSNS) and there were no differences regarding the characteristics of patients undergoing CSNI or CSNS cycles, including the presence of polyps (75.0% vs. 82.4%; *p* = 0.26), except for the lower rate of ex-smokers (6.3% vs. 19.0%; *p* = 0.04*). Only CSNI cycles patients showed significant improvement in the SNOT-22 (44.5–39.7; *p* = 0.04*), as shown in Table 6. On the other hand, only CSNS cycles patients showed significant improvement in LKES (5.4–4.7; *p* < 0.001*).

**Table 6:** Results of CSNI and CSNS treatments for patients never submitted to ESS.

Patients never submitted to ESS		Treatment cycle						Teste	*p*-Value
		CSNI		CSNS		Total			
		n = 244		n = 158		n = 402			
Treatment time (days)	M/SD	150.4	109.9	147.1	73.3	147.9	83.7	T	0.85
Subjective improvement	n (%)	33	68.8	108	76.1	141	74.2	Chi	0.32
Pre-treatment SNOT-22	M/SD	44.5	21.9	31.2	23.3	34.6	23.6	T	<0.001^a^
Post-treatment SNOT-22	M/SD	39.7	22.3	29.4	24.8	32.0	24.5	T	0.008^a^
		Pre-post T	0.04^a^	Pre-post T	0.12	Pre-post T	0.03^a^		
Pre-treatment total LKES	M/SD	5.3	3.0	5.4	3.0	5.4	3.0	T	0.79
Post-treatment total LKES	M/SD	5.2	3.2	4.7	3.1	4.8	3.1	T	0.39
		Pre-post T	0.39	Pre-post T	<0.001^a^	Pre-post T	0.001^a^		
Adverse events	n (%)	0	0.0	0	0.0	0	0.0	Fisher	1.00
Exacerbations	n (%)	3	6.3	11	7.8	14	7.4	Fisher	1.00
Need for subsequent surgery	n (%)	11	22.9	24	16.9	35	18.4	Chi	0.35

Chi, Chi-square test; CSNI, corticosteroid nasal irrigation; CSNS, Topical corticosteroid nasal spray; Fisher, Fisher exact test; M, mean; SD, standard deviation; n, number; %, percentage; LKES, Lund–Kennedy endoscopic score; SNOT-22 (BR), SinoNasal Outcome Test-22 in Brazilian Portuguese; T, T-test.

a p < 0.05.

### Comparison of alternative CSNI therapies (1% compounded budesonide drops vs. betamethasone cream)

When considering the alternative modalities of CSNI (1% compounded budesonide drops and betamethasone cream), 292 cycles of CSNI were computed, divided into 149 cycles of 1% compounded budesonide drops and 143 cycles of betamethasone cream. There was no difference regarding patient characteristics between alternative CSNI therapies, except for a higher prevalence of asthma in the betamethasone cream group (26.9% vs. 38.5%; *p* = 0.03*) and current smoking in the group using 1% compounded budesonide drops (16.8% vs. 7.0%; *p* = 0.01*). Only 1% compounded budesonide drops showed a significant improvement in LKES (5.1–4.6; *p* = 0.02*), with fewer adverse events (0.0% vs. 4.9%; *p* = 0.006*), as shown in Table 7.

**Table 7:** Result of CSNI treatments with 1% compounded budesonide drops and betamethasone cream.

Types of CSNI		Treatment cycle						Test	*p*-Value
		1% Budesonide		Betamethasone		Total			
		n = 149		n = 143		n = 292			
Treatment time (days)	M/SD	158.4	111.8	142.7	81.1	150.7	98.4	T	0.17
Subjective improvement	n (%)	123	82.6	118	82.5	241	82.5	Chi	1.00
Pre-treatment SNOT-22	M/SD	31.8	22.3	33.9	25.1	32.8	23.8	T	0.45
Post-treatment SNOT-22	M/SD	29.6	22.7	32.8	26.8	31.2	24.9	T	0.26
		Pre-post T	0.09	Pre-post T	0.21	Pre-post T	0.06		
Pre-treatment total LKES	M/SD	5.1	3.1	4.4	2.9	4.8	3.0	T	0.04^a^
Post-treatment total LKES	M/SD	4.6	3.2	4.2	3.2	4.4	3.2	T	0.28
		Pre-post T	0.02^a^	Pre-post T	0.19	Pre-post T	0.01^a^		
Adverse events	n (%)	0	0.0	7	4.9	7	2.4	Fisher	0.006^a^
Exacerbations	n (%)	13	8.7	6	4.2	19	6.5	Fisher	0.12
Need for subsequent surgery	n (%)	13	8.7	20	14.0	33	11.3	Chi	0.16

Chi, Chi-square test; CSNI, corticosteroid nasal irrigation; CSNS, Topical corticosteroid nasal spray; Fisher, Fisher exact test; M, mean; SD, standard deviation; n, number; %, percentage; LKES, Lund–Kennedy endoscopic score; SNOT-22 (BR), SinoNasal Outcome Test-22 in Brazilian Portuguese; T, T-test.

a p < 0.05.

Considering only CRSwNP patients undergoing treatment with CSNI, which accounted for the largest number of CSNI cycles (83.2% – 243 cycles, with 119 cycles of 1% compounded budesonide drops vs. 124 cycles of betamethasone cream), this pattern was maintained, with improvement in LKES only in the group treated with 1% compounded budesonide drops (5.6–5.1; *p* = 0.02*) with fewer adverse events (0.0% vs. 4.8%; *p* = 0.03*). The same results were found when considering patients with previous ESS who underwent treatment with CSNI, also a large portion of CSNI cycles (83.6% – 244 cycles, being 119 cycles of 1% compounded budesonide drops and 125 cycles of betamethasone cream): improvement in LKES only in the 1% compounded budesonide group (5.0–4.6; *p* = 0.04*) with a lower rate of adverse events (0.0% vs. 5.6%; *p* = 0.02*).

### Comparison of doses of 1000 µg vs. 500 µg of 1% compounded budesonide drops in CSNI

A total of 72 cycles of CSNI with 1% compounded budesonide drops at a dose of 1000 µg and 66 cycles at a dose of 500 µg were analyzed. Patients with the higher dose (1000 µg) had more comorbidities: ASA intolerance (19.4% vs. 6.1%; *p* = 0.02*) and NSAID intolerance (13.9% vs. 4.6 %; *p* = 0.06). The higher dose (1000 µg) showed better results, with improvement in the SNOT-22 (30.7–27.0; *p* = 0.06) and improvement in LKES (5.5–4.8; *p* = 0.03*), as seen in Table 8.

**Table 8:** Result of CSNI doses with 1% compounded budesonide drops.

Budesonide doses		Treatment cycle with 1% budesonide						Test	*p*-Value
		1000 µg		500 µg		Total			
		n = 149		n = 143		n = 292			
Treatment time (days)	M/SD	161.7	136.3	162.9	837.0	162.3	114.6	T	0.95
Subjective improvement	n (%)	61	84.7	53	80.3	114	82.6	Chi	0.49
Pre-treatment SNOT-22	M/SD	30.7	23.1	32.6	22.1	31.6	22.7	T	0.63
Post-treatment SNOT-22	M/SD	27.0	23.9	32.2	22.3	29.5	23.4	T	0.19
		Pre-post T	0.06	Pre-post T	0.43	Pre-post T	0.10		
Pre-treatment total LKES	M/SD	5.5	2.5	4.8	3.4	5.2	3.0	T	0.20
Post-treatment total LKES	M/SD	4.8	2.9	4.3	3.4	4.6	3.2	T	0.35
		Pre-post T	0.03^a^	Pre-post T	0.08	Pre-post T	0.009^a^		
Adverse events	n (%)	0	0.0	0	0.0	0	0.0	Fisher	1.00
Exacerbations	n (%)	5	6.9	6	9.1	11	8.0	Chi	0.64
Need for subsequent surgery	n (%)	5	6.9	6	9.1	11	8.0	Chi	0.64

Chi, Chi-square test; CSNI, corticosteroid nasal irrigation; Fisher, Fisher exact test; M, mean; SD, standard deviation; n, number; %, percentage; LKES, Lund–Kennedy endoscopic score; SNOT-22 (BR), SinoNasal Outcome Test-22 in Brazilian Portuguese; T, T-test.

a p < 0.05.

## Discussion

The present study is a pioneer investigation in demonstrating the clinical response of CSNI therapies used in Brazil (such as 1% compounded budesonide drops and betamethasone cream) in relation to CSNS, and showed the following unprecedented results: (1) In the overall sample, both CSNI and CSNS improved LKES (4.8–4.4; *p* = 0.01* and 4.6–4.3; *p* = 0.02*), with more adverse events being observed with CSNI ( 2.4%–0.3%; *p* = 0.04*); (2) In the CRSwNP group, both CSNI and CSNS improved the LKES (5.1–4.8; *p* = 0.04* and 4.9–4.6; *p* = 0.03*, respectively). However, in CRSnNP, neither CSNI nor CSNS improved the evaluated parameters; (3) In the group with a previous ESS, only CSNI improved the LKES (4.7–4.3; *p* = 0.01*), in addition to showing a higher rate of subjective improvement and lower rate of exacerbations than CSNS (85.3 % vs. 77.2%; *p* = 0.04* and 6.6% vs. 13.3%; *p* = 0.02*, respectively). In patients who were never submitted to ESS, CSNI improved SNOT-22 (44.5–39.7; *p* = 0.04*), while CSNS improved LKES (5.4–4.7; *p* < 0.001*); (4) In the group treated with CSNI, only 1% compounded budesonide drops improved LKES (5.1–4.6; *p* = 0.02*), with fewer adverse events than in those treated with betamethasone cream (0.0% vs. 4.9%; *p* = 0.006*); (5) Comparing the CSNI doses of 1% compounded budesonide drops, only the higher dose (1000 µg) improved the LKES (5.5–4.8; *p* = 0.03*). In summary, both CSNI and CSNS promoted improvement in the nasal endoscopy, with high rates of subjective improvement and low rates of adverse events and exacerbations in the overall sample and in the CRSwNP sample. There was no improvement with CSNI or CSNS in the CRSnNP group. A previous ESS was the best scenario for CSNI use, which showed better results than CSNS, both in the nasal endoscopy and in subjective improvement and exacerbations. And patients who were never submitted to the surgical procedure was the only scenario in which CSNS showed better results than CSNI regarding the nasal endoscopy improvement. Among the CSNI therapies, 1% compounded budesonide drops showed better results than betamethasone cream, with the 1000 µg dose showing better results than the 500 µg dose.

The treatment of CRS aims to maintain disease control, which presupposes maintaining symptoms at levels that do not cause discomfort to the patient and nasal endoscopy results showing a mucosa close to a healthy status.^1^ Nasal obstruction is usually the most prevalent CRS symptom, with a great impact on quality of life,^12^ data corroborated in the present study, since in both CRSwNP and CRSnNP groups, nasal obstruction was the most prevalent main symptom. Hyposmia as main symptom was more prevalent in CRSwNP, whereas rhinorrhea and facial pain were more prevalent in CRSnNP, which comprise exactly the pattern of symptoms expected in these diseases, as well as the predominance of asthma, ASA intolerance, dipyrone intolerance, and N-ERD found in CRSwNP.^13^ SNOT-22 scores were higher in CRSnNP than in CRSwNP, also in agreement with the literature.^10^

Our patients undergoing CSNI had a more co morbid history, such as intolerance to dipyrone, N-ERD, current smoking and previous ESS. These features are known to be associated with a greater degree of inflammation, with a negative influence on clinical disease control, requiring a more effective clinical treatment.^6^ Our CRS treatment protocol uses a step-by-step algorithm, in which the modalities are used according to patient evolution. Thus, the topical nasal treatment can be initiated with CSNS and intensified to CSNI if clinical control has not been achieved. This would explain the treatment cycles of the CSNI group being applied in potentially more severe patients.

Both CSNI and CSNS seemed to be effective in improving LKES in most settings, although recent studies have shown better results with CSNI than CSNS.^5^, ^6^, ^7^ However, this does not invalidate the treatment of CRS with CSNS, which is already well established and shows a high level of evidence and recommendation.^1^, ^3^ In the present study, treatment with CSNS showed 76.7% of subjective improvement in our patients and fewer adverse events than CSNI, in addition to a low rate of exacerbations (10.7%) and need for surgical procedure after treatment (12.0%). This highlights the need to individualize topical therapy to the degree of disease inflammation: patients with less inflammation could benefit from CSNS, while those with more inflammation might need CSNI. The unrestricted use of CSNI in all CRS patients, from our point of view, may not be cost-effective, as the therapy has higher doses of CS, more adverse events, greater difficulty in preparation and greater chance of non-adherence.^14^ On the other hand, patients with CRS and more inflammation would clearly benefit more from CSNI,^6^ which was corroborated in the present study, with patients with CRSwNP showing better outcomes at CSNI than those with CRSnNP.

Treatment with CSNI showed its greatest advantage over CSNS in the group with a previous ESS, where there was an improvement in the LKES and more evident results in rates of subjective improvement and exacerbations in relation to CSNS. These results are in agreement with the literature, as it is already known that there is greater penetration of the high-volume solution into the operated sinuses and a greater concentration of the medication in contact with the mucosa, promoting better disease control.^9^, ^15^ On the other hand, in patients who were never submitted to the surgical procedure, CSNS showed better results than CSNI in relation to LKES, showing its efficacy as a nasal treatment, and not exactly sinus treatment, as the spray does not penetrate the sinuses.^9^, ^15^, ^16^

The present study is also an unprecedented one in comparing the results of the modalities of CSNI used in our country, 1% compounded budesonide drops and betamethasone cream. The 1% compounded budesonide drops were more effective than betamethasone cream, with fewer adverse events. This 1% budesonide formulation is a liquid one, prepared in 5% glycerin, allowing the easier dilution in saline solution than a dermatological cream, such as betamethasone. Probably, this more homogeneous dilution than that of the cream formulation allows a greater contact of the drug with the affected mucosa, with a higher concentration of the drug, achieving more significant results. Furthermore, the analysis of the doses of 1% compounded budesonide drops showed that higher doses (1000 µg) had better results than lower ones (500 µg). This finding is supported by CSNI studies using higher doses of CS,^5^, ^6^, ^7^ showing that the amount of the drug plays a role in CSNI, and not just to greater sinus penetration due to the high volume.

The SNOT-22 questionnaire is a quality-of-life assessment parameter that often changes after nasal endoscopy.^2^ For this reason, we believe that our results were more significant in the endoscopic assessment than in the symptom assessment. Because our CRS treatment protocol adjusts topical therapy modalities, more patients with difficult-to-treat disease may have been selected in the CSNI group, which is a possible selection bias in our study. Retrospective studies lack the accuracy of the data, but we believe this bias was well circumvented by the fact that our institution’s electronic medical records include mandatory fields containing the data used in this research. Finally, the lack of randomization does not allow robust data in the direct comparisons between treatment modalities.

## Conclusion

Nasal irrigation with high-volume corticosteroids with 1% compounded budesonide drops or betamethasone cream was effective in improving the nasal endoscopic condition in patients with chronic rhinosinusitis, especially in the presence of nasal polyps and in those previously submitted to ESS, in addition to promoting a higher rate of subjective improvement and lower rate of exacerbation than the use of corticosteroid nasal spray in previously operated patients. However, nasal irrigation with high-volume corticosteroids showed more adverse events than corticosteroid nasal spray use.

The use of 1% compounded budesonide drops for nasal irrigation showed better results in the nasal endoscopy with fewer adverse events than betamethasone cream. Moreover, higher doses of 1% compounded budesonide drops (1000 µg) were more effective than lower doses (500 µg).

Finally, corticosteroid nasal spray use was also effective in improving nasal endoscopy in patients with chronic rhinosinusitis with nasal polyps and was more effective than nasal irrigation with high-volume corticosteroid in improving nasal endoscopy in patients who were not previously submitted to surgery.

## Conflicts of interest

The authors declare no conflicts of interest.

## References

[1] Fokkens W.J., Lund V.J., Hopkins C., Hellings P.W., Kern R., Reitsma S. (2020). European position paper on rhinosinusitis and nasal polyps 2020. Rhinol Suppl.

[2] Kosugi E.M., Moussalem G.F., Simões J.C., Souza R.P., Chen V.G., Saraceni Neto P. (2016). Topical therapy with high-volume budesonide nasal irrigations in difficult-to-treat chronic rhinosinusitis. Braz J Otorhinolaryngol.

[3] Orlandi R.R., Kingdom T.T., Smith T.L., Bleier B., DeConde A., Luong A.U. (2021). International consensus statement on allergy and rhinology: rhinosinusitis. Int Forum Allergy Rhinol.

[4] Rosenfeld R.M., Piccirillo J.F., Chandrasekhar S.S., Brook I., Ashok Kumar K., Kramper M. (2015). Clinical practice guideline (update): adult sinusitis. Otolaryngol Head Neck Surg.

[5] Steinke J.W., Payne S.C., Tessier M.E., Borish L.O., Han J.K., Borish L.C. (2009). Pilot study of budesonide inhalant suspension irrigations for chronic eosinophilic sinusitis. J Allergy Clin Immunol.

[6] Snidvongs K., Pratt E., Chin D., Sacks R., Earls P., Harvey R.J. (2012). Corticosteroid nasal irrigations after endoscopic sinus surgery in the management of chronic rhinosinusitis. Int Forum Allergy Rhinol.

[7] Harvey R.J., Snidvongs K., Kalish L.H., Oakley G.M., Sacks R. (2018). Corticosteroid nasal irrigations are more effective than simple sprays in a randomized double-blinded placebo-controlled trial for chronic rhinosinusitis after sinus surgery. Int Forum Allergy Rhinol.

[8] Wormald P.J., Cain T., Oates L., Hawke L., Wong I. (2004). A comparative study of three methods of nasal irrigation. Laryngoscope.

[9] Harvey R.J., Debnath N., Srubiski A., Bleier B., Schlosser R.J. (2009). Fluid residuals and drug exposure in nasal irrigation. Otolaryngol Head Neck Surg.

[10] Kosugi E.M., Chen V.G., Fonseca V.M., Cursino M.M., Mendes Neto J.A., Gregório L.C. (2011). Translation, cross-cultural adaptation and validation of SinoNasal Outcome Test (SNOT): 22 to Brazilian Portuguese. Braz J Otorhinolaryngol.

[11] Lund V.J., Kennedy D.W. (1997). Staging for rhinosinusitis. Otolaryngol Neck Surg.

[12] Snidvongs K., Heller G.Z., Sacks R., Harvey R.J. (2014). Validity of European position paper on rhinosinusitis disease control assessment and modifications in chronic rhinosinusitis. Otolaryngol Head Neck Surg.

[13] Huvenne W., van Bruaene N., Zhang N., van Zele T., Patou J., Gevaert P. (2009). Chronic rhinosinusitis with and without nasal polyps: what is the difference?. Curr Allergy Asthma Rep.

[14] Jin J., Sklar G.E., Min Sen Oh V., Chuen Li S. (2008). Factors affecting therapeutic compliance: a review from the patient’s perspective. Ther Clin Risk Manag.

[15] Grobler A., Weitzel E.K., Buele A., Jardeleza C., Cheong Y.C., Field J. (2008). Pre- and postoperative sinus penetration of nasal irrigation. Laryngoscope.

[16] Snidvongs K., Chaowanapanja P., Aeumjaturapat S., Chusakul S., Praweswararat P. (2008). Does nasal irrigation enter paranasal sinuses in chronic rhinosinusitis?. Am J Rhinol.

